# Smell Is Emotion

**DOI:** 10.3390/brainsci16010059

**Published:** 2025-12-31

**Authors:** Rachel S. Herz

**Affiliations:** Department of Psychiatry and Human Behavior, Warren Alpert Medical School, Brown University, Providence, RI 02912, USA; rachel_herz@brown.edu

**Keywords:** olfaction, odor, smell, emotion, hedonic valence, mood, feelings, affective science

## Abstract

This perspective piece discusses various facets of affect interpreted through the sensory modality of olfaction. Through a review of the terms “emotion”, “hedonic valence”, “mood” and “feelings” with theory, neurobiology and empirical evidence, I suggest the provocative argument that smell and emotion are fundamentally equivalent and that the essence of olfactory experience is emotion. It is hoped that this perspective piece will help broaden definitions and understanding in affective science, and inspire further research, and theoretical developments in olfaction, emotion and related clinical practices.

## 1. Introduction

Haynes-LaMotte points out in his proposition for a new comprehensive framework of emotion that there is currently no generally accepted consensus on how “affect” or its outputs are defined, in spite of the topic engendering a very large corpus of interdisciplinary research and clinical sciences [1]. Building on Haynes-LaMotte’s comment, I believe that a general underlying conceptual framework for affect has great utility, but it is also the case that granular perspectives offer insights and opportunities that propel empirical and theoretical development in novel directions. In this perspective article I aim to define how various facets of affect are represented and experienced through the lens of olfaction to aid in the evolution of affective science.

**Definitions and Limitations**: For the purposes of this article where a noun is used, the terms “smell”, “odor”, “scent”, “fragrance” and “aroma” all refer to the conscious perception of a volatile organic compound (VOC). “Olfaction” is the sense of smell. 

The affective terms to be defined for this special issue that I will address are: emotion, mood, and feeling. To this list I add “hedonic valence,” as it is a specific aspect of affect elicited by olfactory perception. Most simply and in the context of olfaction, these affective terms are described as follows: emotions, moods and feelings are physiological and psychological states involving the intersection of various degrees of arousal and pleasure. Emotions are discrete and time delimited. Moods are more generalized and longer lasting than emotions. Feelings represent a sense of prior experience either concretely with an odor or abstractly in emotional space. Hedonic valence refers to the qualitative factors individually and in combination that produce basic approach-avoid responses. Each of these terms are explored and discussed in detail below. 

Other olfaction scientists may have different viewpoints from my own, nonetheless, I hope to capture some fundamental features that will resonate across the field. This article presents a high-level analysis from my perspective, rather than a systematic review or deep dive into each of the areas of olfaction and affect that are discussed here. As such, only a subset of relevant literature in each topic area is covered and I apologize in advance to the researchers whose work is not included. The interested reader is referred to the many comprehensive reviews and original research papers on olfaction and emotion that are available. 

## 2. Emotion

At its most essential level, smell is emotion. There are several reasons for my assertion. 

First, neurobiologically, the primary olfactory cortex, which includes the piriform cortex, olfactory tubercle, entorhinal cortex, hippocampus and amygdala is where the conscious perception of smell originates [2,3,4]. It is of central importance that the amygdala and hippocampus are key interconnected regions of the primary olfactory cortex [4]. The amygdala is the neural locus of emotional processing [5,6]. Thus, our aware perception of scent and our experience of emotion are neuroanatomically interconnected and interactive. The hippocampus is the neural area responsible for various forms of association, learning, memory and spatial representation [7], and as will be explained below associative learning and memory are fundamental to the perception of scent. No other sensory system is as neurobiologically coupled with the brain regions responsible for emotional processing, learning and memory as the sense of smell [8,9]. This neurobiological privilege is why the sense of smell is uniquely emotional and founded in the neuroplasticity of associative learning and memory [10,11,12,13,14].

Second, the span of mental states that both emotional experience and olfactory perception encompass are largely equivalent ranging from general and vague appraisals of pleasantness, familiarity, and liking, to full blown explicit, specific and complex emotions [12,13,14,15]. One can feel generally happy or one can feel a deeply specific sense of bliss and fulfillment. Likewise, one can smell a perfume and perceive it as pleasant, or one can smell a perfume and experience a flood of grief because it was the scent worn by one’s recently deceased best friend. For reviews on the psychological parallels between emotion and olfaction see Soudry and Herz [8,16]. 

Third, from an evolutionary perspective, the neural tissue that through phylogenetic development became the specific regions of the limbic system and govern different discrete operations (e.g., amygdala, hippocampus, hypothalamus) originated from cells that were solely dedicated to processing chemical stimuli. This is known as “the chemical brain hypothesis” [17]). These critical survival signals enabled determining and distinguishing chemicals that were good to eat, chemicals that were toxic, chemicals that signaled a reproductively available conspecific, and chemicals that indicated one would be annihilated [18]. Life or death is the most fundamental driver for affect. Therefore, although other sensory systems with inherent affective qualities, such as touch, are highly interconnected with limbic processing, the origins of the limbic system do not appear to be based on somatosensation apart from the connection between somatosensation and chemical sensing [19,20]. 

Additionally, from a functional perspective, emotions aid our survival in a similar manner that the basic positive and negative survival signals imparted by chemical stimuli do. Positive emotions lead to actions that support going toward the emotion inducing stimulus enabling safety, security and reproduction. Negative emotions lead to actions that support moving away from or eliminating the source of the stimulus and help us escape, combat, and withdraw from threats. Correspondingly, odors that we perceive as smelling good encourage us to move closer to the source of the odor, and odors that we perceive as smelling foul encourage us to move away from the source. 

Based on the primacy of chemical detection in affective evolution, it is suggested that the complex cognition involved in experiencing emotions originated from the perception of chemical stimuli (i.e., odors) that provided positive and negative information essential to basic survival [21]. I have proposed that the human emotional system is a highly evolved, abstract cognitive version of the basic behavioral motivations instigated by the olfactory system in non-human animals [22]. Smell for most animals informs survival in direct and explicit ways. In humans, olfactory signals are not the primary data used for survival, and among the sensory systems vision is dominant. Instead, olfactory survival codes have been transformed into our experience of emotions. That is, the human brain has co-opted the survival-guiding chemical sensing systems of other animals into our survival-guiding system of emotions [22]. 

Finally, from a clinical perspective, acquired anosmia (complete smell loss) is correlated with substantially altered emotional experience. Individuals who lose their sense of smell tend to experience much higher levels of depression, social dysfunction and decreased quality of life [23]. These emotional disturbances are due to the reduced activation and dysregulation of the amygdala and hippocampus that result from the loss of olfactory input and stimulation [24,25,26]. The relationship between depression and olfactory loss is also reciprocal [27]. Individuals going through an episode of severe depression exhibit reduced olfactory function which recovers to normal levels when the depressive episode resolves or is successfully treated pharmacologically [28,29]. Correspondingly, individuals with olfactory dysfunction experience symptoms of depression that worsen with the severity of smell loss, and when they regain their sense of smell, a reduction in depressive symptoms simultaneously occurs [30]. In sum, the sense of smell and emotional experience are fundamentally interconnected, bi-directional, and functionally equivalent [21,22].

Innate responses are biologically hardwired and relatively universal, whereas learned responses are acquired, malleable, context dependent and individualistic. The modifier to the correspondence between emotion and olfaction, is that among healthy humans the basic emotions of happiness, fear, anger, and sadness appear to be largely innate, present from a very early age, and automatically elicited by prototypical stimuli [31,32,33]. By contrast, our responses to scents are chiefly learned through experience and vary by context, subjectivity, personal history, and genetics as explained further below and in the Section 3 [2,34,35,36].

Our emotional response to a scent is not due to its chemical make-up per se (i.e., not due to the specific VOCs that create the perception), but rather the individual’s subjective perception of the scent. That is, it is not the chemical specificity of the stimulus, but the perception of it that matters. If I don’t perceive the scent of a large striped cat with gnashing teeth as a threat, I won’t feel fear. In vision, however, unless you are the Tiger King, seeing a tiger roaring its jaws at us will instantly provoke fear. 

There are several factors that explain why subjectivity plays such a large role in olfactory perception. For one, the chemicals involved in an odor compound can vary greatly and yet yield a consistent perception. For example, the single VOC phenylethyl alcohol elicits essentially the same pattern of neural activation as the more than 1000 VOCs that are present in the living rose bush. Second, as detailed below, we each have a unique nose due to both our genetics and our personal history and this filters all of our responses to chemical stimuli [37].

### 2.1. Genetic Variability

The olfactory gene family in humans comprises approximately 1000 genes, however each of us express only between 350-400 of these genes as functional ORs [38]. The specific ORs that are expressed and how many copies of each receptor type that are functional is highly variable across individuals. Indeed, in genetic terms, no two people (except identical twins with equivalent epigenetic influences) have the exact same complement of ORs [39,40]. This translates to our susceptibility to form positive or negative hedonic associations. For example, if you possess the non-functional gene for detecting the herbal, floral component of cilantro and only detect a soapy quality, which tends to be disliked in food, this will predispose you to disliking cilantro aroma. By contrast, if you possess the full complement of ORs for detecting the herbal-floral cilantro bouquet this will lead to a higher potential for appreciating this aroma in food. Additionally, the number of ORs of a particular type that are expressed modulates liking for an odor. The more receptors that are activated by a specific VOC the more intense that VOC will be perceived to be [41,42]. High intensity stimulation in olfaction, as in all our senses, tends to be less pleasant than moderate or lower level intensity. Therefore, someone with fewer ORs tuned to a particular VOC will be more readily predisposed to approach that VOC. 

### 2.2. Subjective Variability

Our past experience with a scent and the meaning that we have learned to it powerfully drives odor hedonics and is the basis for further complex emotional responses. For example, if you first smelled a diluted mixture of sodium hypochlorite at your childhood swimming lessons, then you may call the scent “swimming pool” and have positive associations and emotional responses to it. However, if you first smelled it while on your knees cleaning a dirty toilet, then you may to call it “bleach” and have negative associations to it. And if you’ve never smelled sodium hypochlorite you would not have any prior association and therefore would most likely respond neutrally, though depending upon the intensity of the solution and the degree of trigeminal activation (e.g., burn, irritation) it elicits, you may experience pain which would lead to withdrawal (dislike, unpleasantness) in the moment. 

Subjectivity in odor perception is also widely observed at a cultural level. For example, in the United Kingdom, methyl salicylate is a common scent in toilet cleaning products and medical balms and thus tends to be associated with unpleasant tasks and circumstances and consequently is rated very low in pleasantness [43]. By contrast, in the United States methyl salicylate is typically experienced in gum and candy combined with the taste of sweetness. Sweet taste is innately positive and treats are rewarding. Thus, pleasantness ratings for methyl salicylate in the US are high [44]. Complex interactions between genetic and subjective factors are also often present. For example, if someone grew up in the US and knew methyl salicylate only from sweet treats but had many receptors that responded to this VOC, and therefore perceived wintergreen as extremely strong, they will likely find the scent unpleasant because of its aversive intensity, even though it is subjectively associated with positive attributes. 

The reason that human scent responses are subject to such variation and neuroplasticity is based in evolutionary principles. Humans are generalists—species that live throughout the varied ecosystems of the earth and have diverse prey and predators. It is thus maximally adaptive that the situation in which an odor is encountered shape the responses that the scent subsequently elicits. For example, the same scent could signify food in one habitat, and poison in another. By contrast, for animals that are specialists and live within restricted ecological niches with specific prey and predators, such as the panda bear, it is most adaptive that the meanings of scents are innate so that they can most efficiently respond to danger, mates, food (e.g., bamboo) and so on, and they are born with an innate response to the scents of their natural predators and food. This pattern of acquired versus hardwired responses to odors is seen across generalist and specialist species [45,46]. Note that, unlike olfaction, taste responses are largely innate due to the critical survival meaning of the macronutrients that the percept signals. For example, sweet taste is innately pleasurable due to sweet being the signal for carbohydrates, which are the easiest form of convertible energy needed for survival and hence inherently appetitive. For a review of taste perception and hedonics see Mennella 2014 [47].

With regard to debates between primary and secondary emotions, in olfaction whether the emotion represents a primary basic emotion or a complex secondary emotion is irrelevant. There are various definitions and distinctions between primary and secondary emotions in the affective sciences literature. I will take the Damasio (1994) position that primary emotions are innate, universal, fast acting, non-uniquely human, and enable adaptive responses to survival based stimuli; for example, fear, sadness, anger, joy [48]. By contrast, secondary emotions require more cognitive reasoning and learning, and are typically based on inferred social appraisal; for example, relief, hope, guilt, confidence. However, regardless of how primary or secondary emotions are defined, the point in olfaction is that it does not matter what type of emotion is elicited by an odor, simply that an odor can elicit any emotion to which is it associated and understood to mean. If a certain fragrance is associated to feelings of power and confidence and interpreted thusly then those secondary emotions will be elicited when the scent is smelled. If, instead, the same fragrance is associated with the loss of a loved one, and interpreted as such, then that primary emotion of sadness will be elicited when the scent is encountered. The critical point is simply that any emotional state that is elicited by an odor is due to the meaning of the odor for the individual at the time of odor exposure. See the Section 3 for further discussion.

With respect to the duration of emotions elicited by odors, I take the position that emotions are bounded in time in relation to the presence of the stimulus eliciting the emotion [49]. That is, the emotion generally lasts as long as the exposure to the scent or the ability to detect the scent lasts. Odor adaptation (decreased sensitivity) which occurs at the level of the OR will decrease the perceived intensity of a scent within seconds, and a scent can become barely detectable after continuous exposure of approximately 20 min [50,51]. Thus, the duration of emotional responding to a scent is limited by the ability to detect the scent, even if the chemical stimulus remains physically present. 

Additionally, though the aftereffects of specific emotions can linger after the stimulus is no longer present, the emotion typically morphs into a different emotional state. For example, suddenly encountering a bear while hiking alone in the woods likely induces an intense state of fear. But when the bear loses interest and ambles away, though there may still be some residual fear, the predominant emotion becomes giddiness and/or relief. In other words, it is specifically within the presence of the stimulus (bear) that the emotion (fear) is felt. Similarly, it is specifically while perceiving the cologne of one’s high-school sweetheart that one feels an emotional response. When not in the presence of the cologne the emotion is not elicited. Though a general mood such as nostalgia from having smelled the scent may persist beyond the presence of the scent (see Section 4 and Section 5 for further discussion).

### 2.3. Emotion Is Smell

Another level at which “emotion is smell” is that our emotional state changes the odors that our body produces. This is because emotions alter our nervous system physiology, hormonal states, and metabolism and thus change the chemistry of our sweat. These emotional scent changes in our body odor have been shown to be detectable by other humans, as well as a range of species including dogs, cats, and horses [52,53,54]. 

It has also been demonstrated that the perception of emotional body odors can influence the behavior of those who smell them [55]. This has most often been experimentally observed for states of fear, anxiety and stress [56]. For example, smelling the body odor of fear in someone else increases the likelihood of perceiving fear in ambiguous facial expressions [57]. In other words, smelling someone else’s fear readies us for detecting fear among our conspecifics, which could have adaptive value in social settings to prepare the group to respond to a threat [58]. Recent studies have also shown specific body odor changes and responses to the scents of sexual arousal and aggression. Hoffmann found that the smell of sweat from sexually aroused women in their luteal phase (during which time conception is possible) increased male sexual arousal compared to the smell of sweat from the same women in a non-emotional state [59]. Agron demonstrated that when men were exposed to the scent of women’s emotional tears of sadness, their experimentally manipulated aggression dropped significantly compared to being exposed to saline alone (water and salts, i.e., saline, is the major non-emotional components of tears) [60]. Thus, our emotional state is manifested in how our body’s smell, these odors are detectable, and the scent of our emotions influences others in an emotionally adaptive manner.

## 3. Hedonic Valence

Hedonic valence is the first level of affective experience. In olfaction, hedonic perception is captured by the dimensions of pleasantness, familiarity and intensity and is traditionally evaluated with subjective rating scales. Together these dimensions constitute an experience on the continuum of positive-negative. Hedonic responses which are rooted in simple approach-avoid actions, translate to liking-disliking [45,61]. 

‘Liking/disliking’ and ‘positivity/negativity’ are affective states that generate concordant behavioral responses (approach or avoid). As mentioned above, hedonic responses to odors are predominantly learned, not innate. Though, it is noted that disproving the involvement of innate influences in odor hedonic perception is difficult.

Among humans, without any prior experience with a specific scent, the first response is either non directional or cautiousness-- the adaptive neophobic response to approach warily until meaning has been determined. Once the meaning of a scent has been discovered, associative learning governs the emotional and hedonic responses to that odor that follow. For example, an aroma that is paired with a pleasurably sweet food will lead to liking and subsequent approach. Alternately, if this scent is paired with a rotted food it will be learned to be disliked and avoided. 

Hedonic valence as a general appraisal can also evolve into more intense, complex and divergent emotional responses and those responses can change with the meaning of the scent to the perceiver over time. For example, I originally smelled a fragrance when I was a teenager worn by my first love, and I adored it because it signified my love. Then, a few months later, I suffered a very humiliating break-up with this person and the fragrance came to signify my chagrin. Fast forward 10 years and I smell that scent by someone passing me on the street and I am warmed by nostalgia and the reminiscence of my youthful folly. The critical point is that the scent is the same in each case, but the meaning has changed as a function of my current perspective, and thus the hedonic valence of my response to it (liking-disliking-liking) varies accordingly. 

Associative learning explains the bulk of olfactory hedonic valence responses, however, there are several qualifiers and caveats:

(1) Odor intensity: Perceived odor intensity is affected by (A) individual differences in the ORs that each individual expresses, which modifies chemical detection and intensity perception as described previously. And (B) Proximity to the source. If someone is excessively spraying themselves with cologne the intensity of the scent is extremely high to those nearby and therefore may be much less pleasant to others than the same fragrance at moderate intensity. The relationship between odor intensity and odor pleasantness is dependent on the specific odor perceived [62], but generally follows an inverted-U function for prototypical pleasant odors and a linear function for prototypical unpleasant odors [37].
(2) Context effects: Once the meaning of an odor has been learned, the corresponding hedonic responses will be elicited when that odor is encountered again, as long as the odor is interpreted in the same way. However, if the environmental or situational context alters odor interpretation, hedonic responses will be altered accordingly [63]. For example, a certain aroma in a French restaurant is likely to be perceived positively and as signifying the proximity of cheese, but the same chemical mixture in a dark alley is most likely to be perceived negatively and as indicating the proximity of vomit or garbage [64].
(3) New associative learning, when later exposure to the same scent occurs but with a very different meaning. This is most often seen when subsequent exposure is associated with very negative emotional experiences. For example, the smell of BBQing meat which was found pleasing from positive experiences of eating and grilling meats, becomes abhorrent after experiencing a very similar aroma in a traumatic situation. The smell of burning flesh among mammals is very similar as a function of the Maillard reaction [65]. For example, no qualitative VOC differences between burned pig and human cadavers were found using GC-MS [66]. If an individual who likes the aroma of grilling meat subsequently encounters the smell of burning human fresh it will become re-associated to the extremely negative event and can be the trigger for a post-traumatic stress episode (see Section 4 for further discussion).
(4) The passage of time: The time lapse between when an original emotional association to an odor was made and intervening life events can substantially alter either or both the detection and the meaning of a scent and hence the emotions that are evoked. This can occur with the changing meaning of a fragrance, as in the cologne example (love, humiliation, nostalgia). It can also occur due to physiological changes in OR receptors or changes to central olfactory function. The most common situation is the loss of ORs due to aging. Depending on which ORs are affected and how many are lost, odor detection and hence hedonic perception can change both qualitatively and quantitatively [67,68].

In sum, the hedonic valence of odors as good or bad is learned and interpreted in context, it is genetically and psychologically variable, and is experienced in time rather than being absolute. This contrasts with most other stimuli that elicit the basic negative emotions (anger, fear, sadness) as they mainly elicit instinctive responses to danger, threat, and loss. The exception in the class of negative emotions is disgust, as the sources of and responses to disgust are (like smell) learned and context dependent [69]. In contrast to negative emotions, there are many varieties of positive emotions elicited by a vast range of sensory, physical and cognitive stimuli that like odors are highly dependent on context and learning [70].

## 4. Mood

I define mood as an affective state that is expressed by the intersection of arousal and pleasure, as proposed by Russell’s affect grid [71]. Moods persist for longer than specific discrete emotions, though duration is not a critical aspect of mood as relates to olfaction. Additionally, moods can encompass several emotions that then convert to a generalized mood. For example, one can feel the specific emotions of guilt and fear which then convert to the mood of anxiety, or the seed emotion of sadness can bloom into the mood of depression.

A discussion of mood in the framework of olfaction most commonly centers around “aromatherapy” effects. Aromatherapy is the colloquial term for the effects of odors on mood and various physical and mental states. It is critical to point out that aromatherapy can be real, but it is not due to pharmacological mechanisms. Rather it is due to the learned associative effects of odors on our emotions as described above [11]. That is, the scent of pine can make you feel invigorated, but only if you have uplifting and energizing associations to this smell. 

Emotions also have downstream influences on our physiology and behavior. Therefore, an odor associated with an exhilarating experience (e.g., pine trees) can increase your heart-rate and may also make you run faster. However, if you have never smelled pine before, or if you dislike the scent, it will produce either no emotional effects or negative states [11].

Another indication of the psychological rather than pharmacological mechanisms underlying odor effects on mood is that they are immediate. The conscious perception of smelling an odor simultaneously evokes an emotional association and that emotional state has immediate downstream effects of our mood, physiology, performance and behavior. If the effect from smelling an odor were pharmacological, the effect would not occur instantly because the odor would need to be metabolized before exerting its influence. 

“Moods” within aromatherapy are almost always positive and range in arousal from uplifting and energizing to calming and soothing. For example, the scent of citrus is alleged to produce an uplifting mood and the scent of lavender to elicit relaxation. However, the purported effects can also be contradictory. For instance, according to at least one compendium of aromatherapy, juniper oil has seventeen different properties ranging from aphrodisiac to sedative [72]. 

Importantly, aromatherapy effects are powerfully bolstered by verbal claims, descriptions and labeling. This is because odors are invisible and as information seeking primates we search for the meaning and source of a stimulus. You can see the pine tree and the scent that is emanating from its needles is its scent. However, when a scent is in a bottle we are totally reliant on being informed by labeling for what that scent is. Thusly, we can be persuaded to believe that a scent is from many different sources unless it is extremely distant from our expectations and prior experience. That is, the degree of divergence between a label and an odor will be a moderator. For example, it would be hard to convince someone that pine aroma signifies that a pepperoni pizza is about to be served, but not hard to convince them that a pine scent is peppermint [63]. 

Even the premise in aromatherapy that “essential oils” are vital to the value and effectiveness of a fragrance is based on marketing. It doesn’t matter whether the aroma is in oil (essential or not, which is a function of the distillation process). What matters is how the aroma is perceived. For example, I have shown that people cannot distinguish between natural and synthetic versions of an odor at above chance levels, and how they respond to an odor and whether they categorize it natural or synthetic is entirely due to their beliefs about the odor’s origin, or the label given to it [34].

The effects of verbal suggestion on olfactory mood effects have been very well documented. In a classic empirical demonstration, participants were exposed to the scents of lavender, neroli or no odor and for each either told that it was “relaxing” or energizing”. Heart-rate and skin conductance as physiological metrics of emotional state were then measured. The results revealed that regardless of what the odor was or even whether an odor was present, when participants believed that the scent was relaxing, their heart-rate and skin conductance decreased, and when they believed it was energizing these same physiological indices of emotion increased [73]. The effect of words on odor perception is classically witnessed in perfume marketing and advertising, where the connotations of a fragrance-- power, seduction, beauty—are created by the brand and the wearer aspires to experience these moods when wearing that perfume [16].

The relationship between olfaction and mood is also bidirectional. Not only can odors influence our moods, our moods can influence our perception of odors. A number of laboratories have shown that anxiety increases odor sensitivity especially for unpleasant odors. For example, Krusemark and Li took advantage of a naturally anxiety producing situation and found that the more anxious a participant felt the more accurate they were at detecting a subthreshold negative odor mixture of trimethylamine and valeric acid (smells like fish and sweaty socks) [74]. Experimentally induced anxiety also causes neutral odors (such as diluted guaiacol—smells slightly smoky-woody) to be rated more negatively than the same odors when rated in a non-anxious state [75], and trait anxiety increases the speed of detection for emotionally valenced odors compared to neutral odors (e.g., diluted rubbing alcohol) [76]. A point of note: describing an odor as “neutral” in valence generally means that hedonic responses to the odor average in the middle of a pleasantness scale or measure rather than towards the end points of that scale or measure within the observed population sample. 

Importantly, outside of aromatherapy, the moods elicited by odors can be negative just as easily as positive, as an extension of the associations and emotions that are connected to a particular scent. For example, a scent that reminded someone of a deceased loved one may produce a mood of depression. Most critically, negative moods induced by odors that trigger a post-traumatic stress response can elicit devastating and long-lasting effects. 

Furthermore, as mentioned in Section 2, affective disorders are often correlated with olfactory dysfunction. Many studies have demonstrated the connection between mood disorders and olfactory abnormalities [77]. Decreased olfactory function has been observed in major depression and bipolar depression [78]. Generalized anxiety disorder has been shown to be correlated with lower scores on various measures of odor perception and sensitivity [79]. Post-traumatic stress disorder has been shown to lead to increased or decreased odor sensitivity depending upon the odor [80]. Even seasonal affective disorder has been shown to produce alterations in olfactory function; in particular an increase in odor sensitivity [81].

## 5. Feelings

A “feeling” within olfaction can be approached from several perspectives. One dimension of an olfactory feeling resembles the ‘feeling of knowing’ state in cognition, where one is confident that one knows something but cannot recall it specifically [82]. This is experienced in olfaction when one feels that an odor is familiar but has no concrete handle on what it is. Indeed, one of the many unusual features of olfaction is that odors can be experienced purely perceptually, without any cognitive semantic overlay [83]. 

In the pure verbal aspect of odor familiarity, this experience is exemplified by the “tip of the nose” state, where borrowing from the classic tip of the tongue phenomenon in verbal memory [84], one cannot recall the name for an odor despite a very strong feeling of ‘knowing’ it [85]. Different from the verbal memory case, however, is that in the tip of the nose state one has no lexical access for the name of the odor in question. Note that familiarity is one of the three dimensions that create odor hedonic valence. Odor pleasantness and familiarity are highly correlated, especially for odors perceived as smelling good [86,87]. Thus, an olfactory “feeling” is inherently imbued with emotionality. 

More germane to the discussion of emotion, another level of “feeling” in olfaction is the sense of nostalgia that scents can evoke [88]. These feelings often don’t have a specific cognitive source and the affective states they elicit are diffuse, vague, and generally positive. The reason why odors frequently trigger nostalgic feelings is because many first associations with odors take place in childhood [89], and childhood is typically the event horizon from which nostalgia arises [90]. Interestingly, it is also frequently the case that the nostalgic feeling that is elicited by an odor is evoked by odor that one cannot verbally access [83]. Thus, the cognitive feeling of the tip of the nose state and the emotional feeling of nostalgia often overlap in olfaction. Another conjoined level of both familiarity and nostalgia is when odors elicit feelings of déjà vu-- where the presence of an ambient scent alters a scene such that one has the feeling of having been in the same space and/or state before. The sensation of déjà vu is also processed in the amygdala, again demonstrating the conjunction of olfaction with the varieties of affective experience [91]. 

## 6. Conclusions

This article presents my analysis of emotion through the lens of the sensory system of olfaction. Through a review of the neurobiology, evolutionary significance, functional purpose and conscious experience of olfaction, I have argued that the sensory perception of odors and the experience of emotion are fundamentally the same. I have explained this proposition by defining the terms “emotion”, “hedonic valence”, “mood” and “feelings” with evidence and theory that illustrate the inherent convergence between odor perception and emotional experience. It is my hope that a better understanding of the intersection of olfaction and emotion will provide a more complete understanding of emotional states such that different levels (and terms) of emotion can be more fully defined within a general underlying conceptual framework of affect, as well as an expanded granular analysis of affect. Additionally, I hope that articulating a provocative argument—smell is emotion—will inspire future research and theory in olfaction and propel new developments in clinical and affective science.

## Data Availability

No new data were created or analyzed in this study.
